# Therapeutic synergism: How can psychopharmacology improve cognitive rehabilitation?

**DOI:** 10.1590/1980-57642018dn13-040009

**Published:** 2019

**Authors:** Leonardo Caixeta, Victor Melo Caixeta

**Affiliations:** 1 Universidade Federal de Goiás Faculdade de Medicina - Neurology, Goiânia, GO, Brazil.; 2 Federal University of Goiás Faculty of Medicine Ringgold standard institution - Post-Graduation, Goiânia, GO, Brazil.

**Keywords:** cognitive rehabilitation, treatment engagement, psychopharmacology, synergism, reabilitação cognitiva, tratamento, psicofarmacologia, sinergismo

## Abstract

Despite recent advances in cognitive rehabilitation of patients with cognitive disorders, there are many major obstacles to the optimized global use of this therapeutic resource.

**Objective:**

The authors outline the concept of ‘therapeutic synergism’, i.e. the concurrent use of pharmacological and cognitive rehabilitation therapies to maximize functional benefits, addressing the optimization of therapeutic approaches for cognitive disorders.

**Methods:**

Three psychopharmacological and rehabilitation interrelationship paradigms are presented in three different clinical settings.

**Results:**

Paradigm 1: Behavioral and cognitive symptoms that hinder a cognitive rehabilitation program, but can be improved with psychopharmacology. Paradigm 2: Cognitive symptoms that hinder cognitive rehabilitation, but can be improved with anticholinesterases. Paradigm 3: Behavioral symptoms that hamper the use of cognitive rehabilitation, but can be improved by psychotropic drugs.

**Conclusion:**

Judicious use of psychotropic drugs in cognitive disorders can benefit, directly or indirectly, cognitive functions, thereby favoring other treatment modalities for cognitive impairment, such as neuropsychological rehabilitation.

## RESUMO

Apesar dos recentes avanços na reabilitação cognitiva de pacientes com distúrbios cognitivos, existem muitos e graves obstáculos ao uso otimizado globalmente desse recurso terapêutico.

**Objetivo:**

Os autores destacam o conceito de ‘sinergismo terapêutico’, ou seja, o uso simultâneo de terapias de reabilitação farmacológica e cognitiva, maximizando os benefícios funcionais, a fim de abordar a otimização da abordagem terapêutica dos distúrbios cognitivos.

**Métodos:**

Três paradigmas de inter-relacionamento psicofarmacológico e de reabilitação são apresentados em três contextos clínicos diferentes.

**Resultados:**

Paradigma 1: sintomas comportamentais e cognitivos que dificultam um programa de reabilitação cognitiva, mas podem ser melhorados com a psicofarmacologia. Paradigma 2: sintomas cognitivos que dificultam a reabilitação cognitiva, mas podem ser melhorados com anticolinesterásicos. Paradigma 3: sintomas comportamentais que dificultam o uso da reabilitação cognitiva melhorada por drogas psicotrópicas.

**Conclusão:**

O uso criterioso das drogas psicotrópicas nos distúrbios cognitivos pode beneficiar, direta ou indiretamente, as funções cognitivas, favorecendo, portanto, outras modalidades de tratamento para o comprometimento cognitivo, como a reabilitação neuropsicológica.

Despite recent advances in cognitive rehabilitation of patients with cognitive disorders, there are many major obstacles to the optimized global use of this therapeutic resource. Some patients may find it difficult to adhere to cognitive rehabilitation due to the lack of insight regarding cognitive deficits, or because of compromised brain systems, or even general difficulty in performing daily tasks, sense of hopelessness, lack of energy and apathy, symptoms that may be due to the disease itself or associated to depression or other comorbid psychiatric disorders.^1^ For cognitive rehabilitation methods to be effective, patients must be adequately engaged and motivated not only to begin a rehabilitation process, but also to remain involved in the intervention until a therapeutic dosage can be achieved.^1^ Many patients do not benefit from rehabilitation or cannot be indicated for this procedure because of partially treated behavioral symptoms, either for lack of a diagnosis, or for inadequate or underdosing of medications. On the other hand, misuse of drugs by patients, especially medications with cognitive effects, can compromise the efficacy of cognitive rehabilitation.^2^

Notwithstanding recent evidence suggesting that concurrent pharmacological and behavioral methods may maximize functional benefits for patients suffering from, for example, dementia,^3^^-^ 5 there is an inexplicable scarcity of studies concerning the therapeutic synergism between psychopharmacology and cognitive rehabilitation, reflecting the unfortunate absence of contact between these two domains: pharmacological (biomedical approach) and non-pharmacological (essentially the psychological approach). 

In this article, we present three paradigmatic cases on how these two domains can be interconnected and through which strategies the psychopharmacological approach can optimize the implementation of cognitive rehabilitation techniques to enhance improvement in real-world functioning.

## METHODS

Using a qualitative approach, the main principles or strategies of association between psychoactive drugs and cognitive rehabilitation used in the Memory Clinic at the Federal University of Goiás (UFG), in Central Brazil, were reviewed. We focused attention on pharmacological management that addresses the optimization of cognitive rehabilitation techniques.

The Institute of Memory at the UFG is a referral center for cognitive disorders in Central Brazil with 20 years of experience in the evaluation, diagnosis and multidisciplinary treatment of cognitive disorders. Centers such as this have academic credentials to seek, through their accumulated experience, some subjective principles that govern conduct and therapeutic strategies in areas where there is little literature.

## RESULTS

We identified three models of interaction between psychoactive drugs and cognitive rehabilitation that seek to optimize rehabilitation methods. Each of these models will be exemplified by a clinical case illustrating the way psychopharmacology and cognitive rehabilitation interact.

### CASE 1

**Paradigm 1:** Behavioral and cognitive symptoms that hinder a cognitive rehabilitation program, but can be improved with psychopharmacology

Cloney (fictitious name), 25 years old, is a patient with invasive developmental disorder (autistic syndrome), presenting with severe behavioral and cognitive changes. The behavior alterations that prevented a cognitive rehabilitation approach included impulsivity and aggressiveness (he took the papers from the teacher’s desk and ripped them up compulsively, despite being asked not to do so several times; whenever a child passed him by, he would grab them by the arm and attack them; he presented several episodes of direct violence towards people who assisted him; disobeyed commands and did not respect the social limits and rules previously imposed), dysphoria, compulsive overeating, motor restlessness and hyperactivity. Cognitive disorders included severe mental retardation, with impairments in several cognitive domains. The cognitive disorders that most affected his functional adaptation and social life were cognitive inflexibility, insight absence, Theory of Mind deficit, impaired decision-making, expressive language difficulties, and severe attention deficit. Cloney also presented extreme intolerance to any modification of his environment, reacting aggressively when this occurred (he broke everything around him).

Due to prejudice held by both his mother and the multi-professional care team, Cloney was not taking any medication for his disorder. After explaining to them how modern psychopharmacy could help him control some of his worse behavioral issues, and maybe even improve some of his basic cognitive functions, thereby allowing a rehabilitation approach, a pharmacological regimen was started consisting of aripiprazole 15mg/day (prescribed to improve social behavior, reduce aggressiveness and control restlessness), fluoxetine 20 mg/day (indicated to control compulsive overeating and dysphoria) and methylphenidate 40mg/day (prescribed to improve attention span, and reduce both hyperactivity level and appetite).

One month after starting on this medication, Cloney showed a marked improvement in many aspects of his behavior, which also presented as benefits in a variety of cognitive functions: 1) motor restlessness ceased and, consequently, he could sit still in a chair, focusing his attention better, allowing a better verbal approach and eye-to-eye interaction; 2) impulsivity also ceased, and his actions became more predictable, improving the safety of the care team since they could prevent certain responses or undesired actions; 3) improved many aspects of his relationships, as he obeyed social rules (started to accept his mother and teachers’ authority) and became more sociable in general, so he could engage in group interactions, including with other children; 4) he became more tolerant to the limits established and to the external rules imposed, eliminating the explosive reactions when his immediate desires were denied; 5) it became easier to negotiate with him on decisions that involved immediate desires - after medication he started to accept that his desires may be satisfied in exchange for some effort (for example, helping his mother or following teachers’ requests). These improvements promoted a better-structured cognitive-behavior base, more amenable to the application of adequate rehabilitation techniques. Before the psychopharmacological intervention, even simple cognitive-behavior approaches were impossible. Currently, Cloney is reasonably engaged in cognitive rehabilitation, and his team of health professionals and teachers, as well as the other students and patients, no longer fear him.

### CASE 2

**Paradigm 2:** Cognitive symptoms that hinder cognitive rehabilitation, but can be improved with anti cholinesterases

Homero (fictitious name), 74 years old, is a patient with Alzheimer’s disease, naïve to treatment with anticholinesterases (most effective medication group for this dementia).^4^ As the treatment with galantamine did not work because of side effects (severe nausea, tachycardia and dizziness), the family were reluctant to try this pharmacological group again. A glutamatergic antagonist (memantine) was prescribed, without any significant benefit in cognition, particularly in memory.

Homero presented a severe memory deficit and inability to learn new information, which made neuropsychological rehabilitation approaches even harder. Despite the three sessions he had every week, there was no effective improvement of the patient, comparing to previous sessions, so it was not possible to advance to the next phases of the process. The family noted no benefits in the social-functional sphere. Since there was no benefit of the neuropsychological rehabilitation, the therapeutic approach was discontinued.

Donepezil (another anticholinesterase, although with more favorable side-effect profile) was then prescribed at the dosage of 10mg/day in order to improve cognitive outcome, especially recent memory and, consequently, enhance his learning mechanisms. Three months after starting use of the medication, Homero presented a clear memory improvement, and his attention capacity was better, favoring the learning process. In fact, the improvement was very evident when he resumed rehabilitation: his attention span had developed, he could maintain recently learned information available for longer in working memory (for example, during the execution of a task, he could gather information that was necessary later for use in a new task). 

### CASE 3

**Paradigm 3:** Behavioral symptoms that hamper the use of cognitive rehabilitation, but can be improved by psychotropic drugs 

Thelma (fictitious name), 67 years old, suffering from depression (not previously diagnosed) associated with dementia in Parkinson’s disease. Despite being in use of an anticholinesterasic drug and memory deficit improvements achieved, her greatest difficulty was adhering to the cognitive rehabilitation. Thelma presented intense fatigability, being incapable of remaining in continuous consultation for more than five seconds. Her low attentional span impaired all the rehabilitation approaches that relied on attention for task execution. She also exhibited economy of effort with many answers like “I don’t know”. She had a pessimistic attitude to cognitive rehabilitation, believing that she couldn’t obtain any benefit from it, and was unable to develop any involvement or affective bonding with the rehabilitation professional, proving averse to the activity. In many situations, she was anxious and irritated with the activity, creating ploys to leave and stop the process.

Thelma was referred to a psychiatrist who diagnosed masked depression. She was started on a noradrenergic antidepressant (mirtazapine 30 mg /day). A month later, clear improvement in the patient’s mood was observed: she was more active and had more physical/mental energy, was more interested in the ongoing tasks, regained the pleasure associated with social contact and other activities, could maintain her attentional focus for much longer, could see the point in investing, more actively, in the rehabilitation. Indeed, after the depression treatment, her involvement and performance in the cognitive rehabilitation activities increased markedly.

## DISCUSSION

From the reported cases, we can infer three models of interaction between psychopharmacology and cognitive rehabilitation:

1. In the patient receiving psychotropic drugs for an underlying disease state where cognitive rehabilitation is indicated in order to improve the residual cognitive deficits associated with the disease (for instance, in schizophrenia, in which the medication is necessary to control the disease, but does not always act on the common cognitive symptoms of this medical condition);^6^^,^ 7
2. In patients with executive dysfunction (one of the areas in which cognitive rehabilitation is known for having more limited results), the use of some drugs can optimize treatment (for example, the use of memantine in patients with cognitive inflexibility, such as in the case of pathological gamblers);^8^
3. Many patients may not be suitable for cognitive rehabilitation due to psychiatric symptoms that hinder the full conducting of the process (e.g. aggressive, agitated patients that are incapable of therapeutic bonding, or apathetic and asthenic patients that do not engage sufficiently in the therapeutic process).^1^^,^ 9 In others, despite attempts to rehabilitate, they have only discrete or diminished improvement because of psychiatric symptoms. In both cases, medication may be used as an agent for reducing dysfunctional behavior, allowing the application of the rehabilitation in a safe and effective manner, or improving results when the rehabilitation is already underway, but in a limited way.

Our study highlights the concept of ‘therapeutic synergism’, i.e. the concurrent use of pharmacological and cognitive rehabilitation therapies maximizing functional benefits in order to address the optimization of the therapeutic approach for cognitive disorders. 

Many other authors have been working on this approach in different scenarios and within different rationales.^5^^,^ 8 ^,^ 10 ^-^ 13 The three paradigms presented describe different scenarios in which a precise drug intervention (precision that must be almost ‘surgical’) helps in the process of cognitive rehabilitation in many ways. 

In the first paradigm, the psychopharmacological intervention must provide the basic conditions to ensure the patient can be indicated for cognitive rehabilitation, otherwise they would not be an eligible candidate.

In the second paradigm, the pharmacological intervention in cognition enables and facilitates the rehabilitation, which may then have a real chance of success. In other words, the prescription of a cognitive enhancer to augment cognitive rehabilitation outcomes, based on a rationale in which a cognitive enhancer proceeds by targeting more basic discrete cognitive skills, so that cognitive rehabilitation can progress to more complex skills. This assumes that the basic skills must be refined before more complex skills can work effectively. Some authors also claim that, currently, cognitive training exercises are used to improve basic cognitive skills, but pharmacotherapy holds promise as a more effective treatment.^5^

In the third paradigm, the pharmacological intervention in behavior optimizes the response to the ongoing rehabilitation process, since it overcomes an obstacle to fully exploit the therapeutic process. For cognitive rehabilitation therapies to be successful, patients must be adequately involved and motivated not only to begin cognitive intervention but also to keep engaged in the rehabilitation program until a therapeutic dosage can be reached.^1^

In a literature review about cognitive rehabilitation, Manzine & Pavarine^14^ found that, in most of the studies reviewed, cognitive rehabilitation can provide more benefit for the patient’s rehabilitation when combined with other interventions, such as pharmacological treatment. Provided that both treatment modalities are aligned and optimized, the synergistic therapeutic effects become evident. To this end, it is fundamental that the attending physician is aware of the whole therapeutic program in which the patient is engaged, having consistent notions of how one treatment modality may impact (positively or negatively) the other, and of how delicate the relationship dynamic is between them. Without such tools, there is a risk of wrongly assessing the risk-benefit ratio involved in each pharmacological choice. In practice, unaware physicians run the risk of prescribing a medication option that may, in some cases, have negative effects on the patient’s cognitive function and also on their cognitive rehabilitation.

In conclusion, judicious use of psychotropic drugs can benefit, directly or indirectly, cognitive functions, thereby favoring other treatment modalities for cognitive impairment, such as neuropsychological rehabilitation. This finding reflects those of other authors.^10^^-^ 12 Fortunately, with better knowledge of the available drugs in general (and psychotropics in particular), greater investment in medical training, as well as a better technical and affective rapport between medical and non-medical professionals, a more optimistic scenario will be possible in the coming years.
